# Fevers in Adult Lupus Patients

**DOI:** 10.7759/cureus.2098

**Published:** 2018-01-22

**Authors:** Homa Timlin, Abrahim Syed, Uzma Haque, Brittany Adler, Genevieve Law, Kirthi Machireddy, Rebecca Manno

**Affiliations:** 1 Medicine, The Johns Hopkins University School of Medicine; 2 Rheumatology, The Johns Hopkins University School of Medicine; 3 Division of Rheumatology, The Johns Hopkins University School of Medicine; 4 Rheumatology, FETCH South Iseland; 5 Other, University of the Sciences In Philadelphia

**Keywords:** lupus, infection, malignancy, vasculitis, fever

## Abstract

Variability in systemic lupus erythematosus (SLE) disease manifestations is well recognized. Lupus disease activity can range from mild to severe. Fever is a common manifestation of SLE and occurs in 36%–86% of patients. In the Modified Systemic Lupus Erythematosus Disease Activity Index (M-SLEDAI), fever is taken into account as disease activity scoring. Assessment of lupus patients with fever is an important diagnostic challenge, since the initial clinical presentation of a patient with lupus is very similar to the acute febrile phase of an infection. The attribution of fever to SLE holds only after other causes are excluded.

## Introduction and background

Normal body temperature is controlled in the thermoregulatory center located in the anterior hypothalamus. Prostaglandin E2 (PGE2) is thought to be the proximal mediator of the febrile response. Elevated levels of intracellular PGE2 in the hypothalamus appear to be the trigger for raising the set point, which then activates neurons in the vasomotor center to commence vasoconstriction and slow the firing rate of warm-sensitive neurons to increase heat production in the periphery. Once the fever is reached, an increase or decrease in core body temperature will stimulate thermoregulatory mechanisms similar to those evoked at normal body temperature to maintain the elevated set point. Thermogenesis in either the fat or muscle takes place by uncoupling proteins, which release adenosine triphosphate (ATP) and heat. Shivering takes place when there is a rapid rise to match the new febrile set point. A morning oral temperature reading >37.2 °C (98.9 °F) or an afternoon temperature >37.7 °C (99.9 °F) is considered a fever [1]. Rectal temperatures are generally 0.6 °C (1.0 °F) higher than oral readings. Fever of unknown origin (FUO) is defined as a temperature higher than 38.3 °C on several occasions and lasting longer than three weeks, with a diagnosis that remains uncertain after one week of investigation [2].

When the hypothalamic set point is reset downward, the processes of heat loss are accelerated through vasodilation and sweating [3, 4]. Pyrogenic cytokines, such as interleukin-1b (IL-1β), tumor necrosis factor (TNF), interleukin-6 (IL-6), interferon alpha (INF-α), interferon beta (INF-β), and interferon gamma (INF-γ) [5-7], are produced by activated macrophages/monocytes and act directly on the hypothalamus to produce a fever response.

Analogous to a biochemical feedback pathway, fever itself appears capable of countering the release of pyrogenic cytokines [4]. SLE patients often have enhanced IFN-α serum levels, and the IFN levels correlate with anti-double stranded DNA (anti-dsDNA) production and disease activity. Furthermore, a close correlation between serum concentrations of INF-α (but not IL-1 or tumor necrosis factor α) and fever was observed in 25 untreated patients with SLE, suggesting the possible involvement of INF-α in fever pathogenesis [5].

IL-1 initiates the recruitment of immune cells and inflammation. Interleukin 1α (IL-1α) and interleukin 1β (IL-1β) are proinflammatory cytokines with widespread biological activities, regulated in part by the interleukin receptor antagonist (IL-1Ra). IL-1 is considered to play an important role in SLE pathogenesis and disease activity. Increased IL-6 and IL-17 serum levels in some studies correlate with elevated anti-DNA levels. Additionally, IFN-γ, as well as T-cell-derived cytokines like IL-21 and IL-2, is dysregulated in SLE. IL-17 induces secretion of many proinflammatory proteins, among them prostaglandin E2 (PGE2), granulocyte-macrophage colony-stimulating factor (GMCSF), granulocyte colony stimulating factor, and also cytokines which induce a positive feedback loop and lead to further IL-17 production like IL-6, IL-1β and IL-21.

## Review

Clinical manifestations

Fever is a common manifestation of SLE and can occur in 36–86% of patients [8-12]. The reported prevalence of fever attributed to SLE has declined progressively, perhaps resulting from frequent use of nonsteroidal anti-inflammatory drugs [8]. Rarely fever may be the only presenting symptom of SLE, as in patients with FUO. Among patients with FUO, up to 5% are eventually diagnosed with SLE [13]. In a large Canadian study, fever typically presented in lupus early in the disease course [14] and is more common in Caucasians. In patients with active SLE without infection, the peak temperature ranges from 38 °C to 40.6 °C with an intermittent pattern.

The differential for fevers in lupus is broad, and includes lupus disease activity, infection, malignancy or drug reactions. Serious infections are a major cause of morbidity among lupus patients and should be considered in all immunocompromised SLE patients with fever. Hence, fever can only be attributed to SLE after other causes are excluded. Rovin, et al. defined an SLE fever with three criteria: 1) absence of infection despite extensive testing, 2) presence of an illness typical of active SLE accompanying the fever, and 3) no evidence for infection despite escalation of immunosuppression [15]. In a retrospective analysis of 160 hospitalized patients with SLE, Stahl, et al. identified 83 febrile episodes in 63 patients [14]. Of these, 23% of the fevers were attributed to infections, 17% to miscellaneous causes, and 60% to lupus disease activity. Inoue, et al. also reported that SLE activity was the most common cause of fever [16].

Compared to patients with SLE and fever of infectious etiology, those with fever due to lupus were more likely to have lower complement C3 and higher levels of disease activity [17]. In addition, fever in the setting of a low white blood cell (WBC) count is more consistent with lupus disease activity rather than infection. A new infection should be strongly suspected if the patient is already receiving moderate or high doses of glucocorticoids and if the fever persists despite other signs of lupus activity remitting [15]. Moreover, a poor response to nonsteroidal anti-inflammatory drugs (NSAIDs), acetaminophen, and low to moderate doses of glucocorticoids should raise suspicion for an infectious or drug-related etiology, since most fevers due to active SLE remit with use of these agents. In an SLE cohort of 22 patients, 11 had SLE fever that was readily suppressed with steroids, unlike the remaining patients with infectious fevers who did not respond well to anti-inflammatory agents [15]. Of note, 10 of these 11 patients with SLE fever had current (class IV) or past lupus nephritis.

Studies have demonstrated fatal sepsis when high doses of steroids are continued in the persistently febrile lupus patient.

Disease activity in lupus

Active lupus can cause fever. Due to the heterogeneity and the fluctuating nature of disease activity, the definition of remission in SLE still remains elusive. A number of SLE disease activity instruments, with overlapping strengths and weaknesses, are available for use. Biomarkers have been added to certain scales including the modified Systemic Lupus Erythematosus Disease Activity Index (M-SLEDAI), the British Isles Lupus Assessment Group index (BILAG-2004), the Systemic Lupus Activity Measure index (SLAM), the Systemic Lupus Activity Index (SLAI), the European Consensus Lupus Activity Measurement index (ECLAM), and the Systemic Lupus Activity Questionnaire (SLAQ) for population studies [18]. Barr, et al. described three major patterns of SLE disease activity over time, as defined by the Physician Global Assessment (PGA) and the modified SLE Disease Activity Index (M-SLEDAI) [19]. The predisposition for flare or remission in the initial years of disease is predictive of long-term outcome, with those achieving remission earlier having a more favorable disease course [20-22]. Stahl, et al. reported that 60% of fevers in lupus were attributable to active SLE [14].

Laboratory findings

Most infectious complications in SLE are bacterial, including respiratory, urinary and soft tissue infections [17,18]. Given the high prevalence of infection in immunocompromised lupus patients, a predictive biomarker that can distinguish infectious fevers from SLE fevers is essential. Present biomarkers have poor specificity and sensitivity for this purpose, but can provide some useful information to help with the clinical assessment for the patient.

Commonly used markers in SLE include anti-dsDNA antibodies, complement (C3 and C4), Erythrocyte sedimentation rate (ESR), C-reactive protein (CRP), anti-C1q antibodies, and activity on urinary sediment. Findings that favor the diagnosis of SLE fever over infection include leukopenia (not explained by cytotoxic therapy), normal or slightly elevated CRP, low C3 and C4, and elevated anti-dsDNA [15]. More specific biomarkers for lupus disease activity are currently being evaluated but are not yet available in clinical practice.

The acute phase response accompanies both acute and chronic inflammatory states and is associated with a wide variety of disorders, including infection, trauma, infarction, systemic autoimmune and inflammatory diseases, and various neoplasms. CRP has both proinflammatory and anti-inflammatory actions [23]. CRP is synthesized by the liver during IL-6 regulation. A major function of CRP is its ability to bind phosphocholine, thereby permitting recognition both of foreign pathogens that display this moiety and phospholipid constituents of damaged cells [24]. CRP can also activate the complement system and bind to phagocytic cells via Fc receptors, suggesting that it can initiate elimination of pathogens and targeted cells by interaction with both humoral and cellular effector systems of inflammation.

CRP increases significantly in SLE patients with concomitant infection, but increases only slightly or not at all in patients with a lupus flare. This has been attributed to the presence of CRP autoantibodies [25], genetic differences in the ability to respond to certain stimuli [26], and reduced production or defects in the action of IL-6 [27]. Furthermore, IFN-α is an inhibitor of CRP promoter activity and CRP secretion [28]. High-sensitivity CRP (hsCRP) is a more sensitive test that can detect very low levels of CRP. HsCRP with values above 6 mg/dl may be associated with active infection with an 84% specificity in SLE patients [29].

The ESR, an indirect acute phase reactant, reflects plasma viscosity and the presence of acute phase proteins, especially fibrinogen. ESR elevation is common in SLE and is often measured as a potential indicator of disease activity. A wide range of factors can influence ESR levels, many of them unrelated to inflammation, such as older age, female gender, anemia, renal disease and technical factors with the assay. Hypoalbuminemia, hypercholesterolemia, hypergammaglobulinemia, and malignancy may also increase ESR. Due to IL-6 secretion by adipose tissue, both ESR and CRP can be elevated in obesity [30].

The ESR is not a variable in the SLE Disease Activity Index (SLEDAI) or the British Isles Lupus Activity Group (BILAG) activity measures, but was included in the Systemic Lupus Activity Measure (SLAM). The ESR/CRP ratio may be useful to differentiate between infection and flare in SLE patients, where a ratio above 15 was significantly correlated with disease activity and a ratio below two was associated with infection [29].

Other causes of fever in lupus

In addition to infection or lupus disease activity, fever may indicate the presence of another concurrent illness, such as sarcoidosis [31], vasculitis [32-35], autoinflammatory disorders [36], rheumatic fever [37], malignancy or drug reaction. Several studies have examined malignancy risk among SLE patients. Mellemkjaer, et al. reported an increased risk of overall malignancy compared with the general population in 1575 SLE patients [38]. This was largely driven by an increased risk for hematological malignancies, particularly non-Hodgkin's lymphoma (NHL). Smedby, et al. found the adjusted odds ratio (OR) for NHL among SLE patients was 4.6 (95% CI: 1.0, 22) [39]. SLE is also a risk factor for cervical neoplasia, and in particular, for premalignant cervical lesions. Malignant disorders can often be differentiated from lupus by their hematological profile, but sometimes bone marrow and/or lymph node biopsy may be needed to confirm the diagnosis. Cancer risk in lupus is associated with a higher cumulative cyclophosphamide dose (OR: 1.09) and lower hydroxychloroquine dose (OR: 0.93).

Vasculitis is also a cause of fever in lupus, and most studies analyzing the prevalence of vasculitis in large series of SLE patients showed a prevalence ranging from 11 to 36%. This vascular inflammatory process may take many clinical forms depending on the size of the affected vessels (arteries, veins, and/or capillaries) and the sites involved (skin or internal organs), with a prognosis that may range from mild to life-threatening [32-35].

## Conclusions

Recurrent fevers in lupus are common and the differential is very broad. Infection should always be considered first and ruled out. If no other clear etiology can be identified, then lupus-associated fevers should be considered.
