# Correction: Fevers in Adult Lupus Patients

**DOI:** 10.7759/cureus.c12

**Published:** 2018-05-01

**Authors:** Homa Timlin, Abrahim Syed, Uzma Haque, Brittany Adler, Genevieve Law, Kirthi Machireddy, Rebecca Manno

**Affiliations:** 1 Medicine, The Johns Hopkins University School of Medicine; 2 Division of Rheumatology, The Johns Hopkins University School of Medicine; 3 Rheumatology, FETCH South Island; 4 Other, University of the Sciences In Philadelphia

The following funding acknowledgement was erroneously omitted from the original published article:

BA was supported by the National Institute of Arthritis and Musculoskeletal and Skin Diseases of the National Institute of Health under Award Number T32AR048522.

